# Effect of immunosuppressants on a mouse model of osteogenesis imperfecta type V harboring a heterozygous *Ifitm5* c.-14C > T mutation

**DOI:** 10.1038/s41598-020-78403-1

**Published:** 2020-12-03

**Authors:** Nobutaka Hanagata, Taro Takemura, Keiko Kamimura, Toshiaki Koda

**Affiliations:** 1 Nanotechnology Innovation Station, National Institute for Materials Science, 1-2-1 Sengen, Tsukuba, Ibaraki 305-0047 Japan; 2 Graduate School of Life Science, Hokkaido University, N10 W8, Kitaku, Sapporo, 060-0810 Japan; 3 Faculty of Advanced Life Science, Hokkaido University, N21 W11, Kitaku, Sapporo, 001-0021 Japan

**Keywords:** Experimental models of disease, Therapeutics

## Abstract

Osteogenesis imperfecta (OI) type V is an autosomal dominant disorder caused by the c.-14C > T mutation in the interferon-induced transmembrane protein 5 gene (*IFITM5*), however, its onset mechanism remains unclear. In this study, heterozygous c.-14C > T mutant mice were developed to investigate the effect of immunosuppressants (FK506 and rapamycin) on OI type V. Among the mosaic mice generated by Crispr/Cas9-based technology, mice with less than 40% mosaic ratio of c.-14C > T mutation survived, whereas those with more than 48% mosaic ratio exhibited lethal skeletal abnormalities with one exception. All heterozygous mutants obtained by mating mosaic mice with wild-type mice exhibited a perinatal lethal phenotype due to severe skeletal abnormalities. Administration of FK506, a calcineurin inhibitor, in the heterozygous fetuses improved bone mineral content (BMC) of the neonates, although it did not save the neonates from the lethal effects of the mutation, whereas rapamycin, an mTOR inhibitor, reduced BMC, suggesting that mTOR signaling is involved in the bone mineralization of heterozygous mutants. These findings could clarify certain aspects of the onset mechanism of OI type V and enable development of therapeutics for this condition.

## Introduction

Interferon-induced transmembrane protein 5 (IFITM5) is a type II (N-in/C-out) transmembrane protein, the amino terminus of which is located inside the cell and the carboxyl terminus is located extracellularly^1^. Although the function of IFITM5 was not known for a long time, it has been suggested for some time now that IFITM5 is a positive regulator of bone formation, as the *Ifitm5* gene is seen to be specifically expressed only in the mineralization stage of cultured osteoblasts^2,3^. Mineralization is promoted and suppressed when *Ifitm5* is overexpressed and knocked down in cultured osteoblasts, respectively^2^. Thus, *Ifitm5*-deficient mice were generated and their phenotype investigated to verify the role of IFITM5 in bone formation. However, studies have neither reported any major abnormalities in bone formation nor morphology in *Ifitm5*-deficient mice^3,4^. Although IFITM5 functions as a positive regulator of mineralization in vitro, it is considered that IFITM5 is not an essential molecule for bone formation in vivo.

Following reports stating that no major abnormality is seen in the osteogenesis of *Ifitm5*-deficient mice, many research groups have reported that patients with osteogenesis imperfecta (OI) type V have a heterozygous point mutation (c.-14C > T) in the 5 ' untranslated region of the *IFITM5* gene^5–16^. The c.-14C > T mutation shifts the start codon of *IFITM5* upstream to create a new start codon, resulting in the addition of 5 amino acids (Met-Ala-Leu-Glu-Pro, MALEP) to the amino terminus. Therefore, OI type V is thought to be caused by the mutated IFITM5 (MALEP-IFITM5) in which these 5 amino acids are added to the amino terminus of IFITM5, however, the mechanism of its onset is unknown at present.

OI is a genetic bone disease characterized by bone fragility, and at least 18 different types of OI are known till date^17^. OI type V is an autosomal dominant condition and is distinguished from other types of OI by features such as forearm interosseous membrane calcification, hyperplastic callus formation, and radial head dislocation. However, the severity of symptoms varies widely among patients. Forearm interosseous membrane calcification is observed in essentially all patients and is associated with a limitation in forearm supination and pronation^5,8–10,18–21^. Hyperplastic callus formation occurs mainly after bone fractures, but is not observed in all patients^5,8–10,18–21^. Radial head dislocation is observed in many patients and causes elbow deformity^5,10,18,19^. There is a remarkable difference in vulnerability to fractures, accompanied by the bone fragility caused by this condition, resulting in variable number of fractures affecting the mobility of patients ranging from being able to walk on their own to needing wheelchairs^9^.

Presently, bisphosphonates are used as therapeutic agents for treating various types of OI including OI type V^9^. The bisphosphonates may increase bone mass by suppressing bone resorption, but improvement of characteristic symptoms of OI type V cannot be expected. The absence of major skeletal abnormalities in *Ifitm5*-deficient mice leads to the possibility that OI type V may be treatable by suppressing the expression of both wild-type (WT) and c.-14C > T mutant *Ifitm5*, which is a similar condition to that in *Ifitm5*-deficient mice.

We previously reported that IFITM5 interacts with the *Fkbp11* gene product (FKBP19), a member of the FK506-binding protein (FKBP) family of peptidyl-prolyl cis/trans isomerases^22^. FKBP19 contains a putative peptidyl-prolyl cis/trans isomerase domain with homology to FKBP12, and FK506 binds to this domain^23^. Therefore, we performed this study to investigate the effect of FK506, an immunosuppressant, on the interaction between IFITM5 and FKBP19. However, it was not possible to investigate this because FK506 suppressed the expression of *Ifitm5*. Meanwhile, the result indicating that the expression of *Ifitm5* was suppressed by FK506 provided us with an important hypothesis, specifically that FK506 might be able to suppress the expression of both WT and c.-14C > T mutant genes of *Ifitm5* in OI type V if the regulatory system for the expression of the *Ifitm5* mutant is the same as that for WT *Ifitm5*. If FK506 could suppress the expression of both the WT and mutant *Ifim5* genes, it can be expected that bone abnormalities will not appear, as in the *Ifitm5*-deficient mice. Therefore, we created heterozygous *Ifitm5* c.-14C > T mutant mice by Crispr/Cas9-based genome editing technology to investigate the effect of FK506 on improving bone abnormalities. This paper presents the bone abnormalities of heterozygous *Ifitm5* c.-14C > T mutant mice, the partial improvement of bone abnormalities mediated by FK506, and the effect of another immunosuppressant, rapamycin, on these bone abnormalities.

## Results

### FK506 suppresses the expression of *Ifitm5* in cultured osteoblasts

As aforementioned, there is a possibility to induce normal bone formation by suppressing the expression of both WT and mutant *Ifitm5* in heterozygote mutant mice.

**Figure 1:**
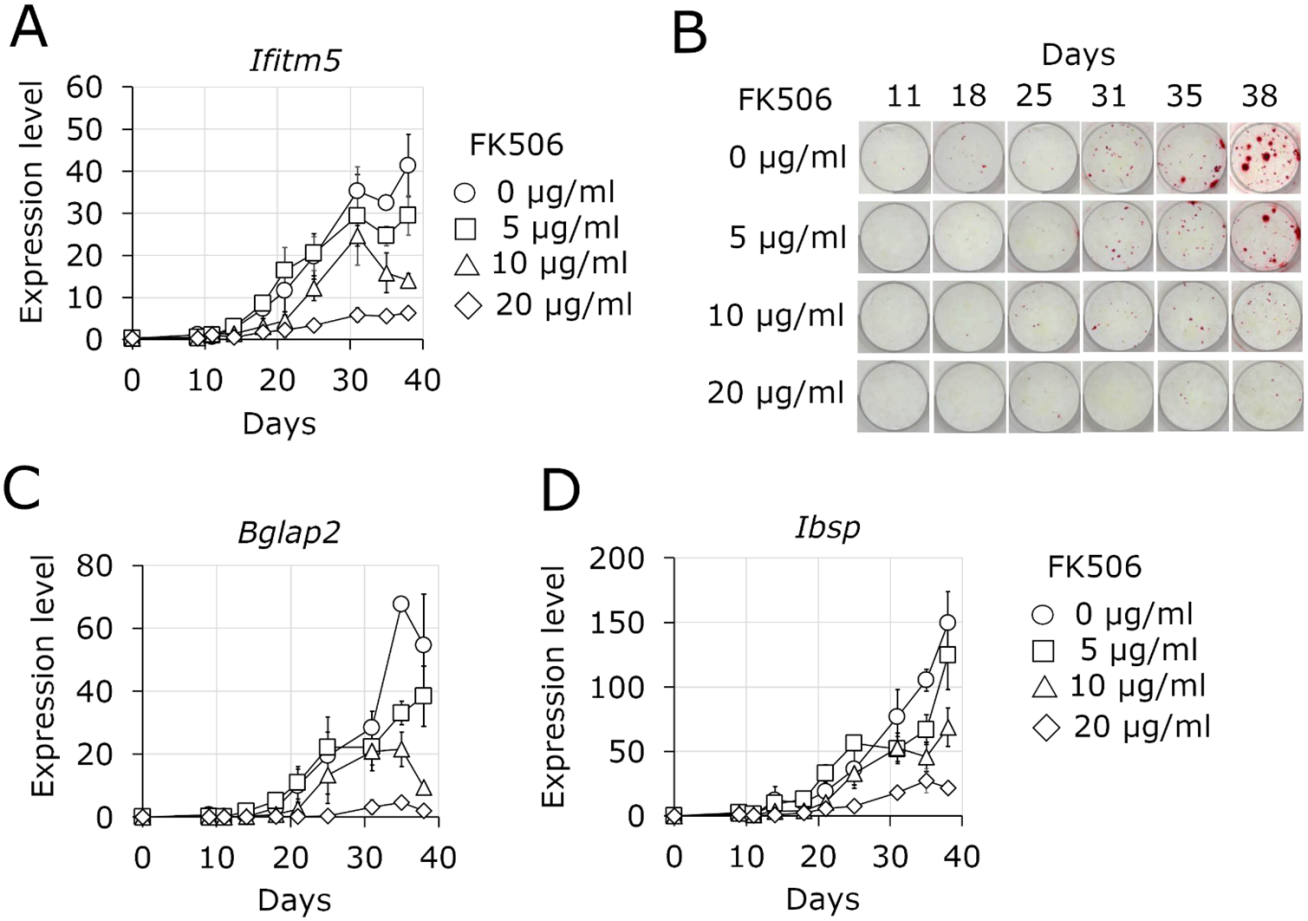
Effect of FK506 on primary mouse osteoblast cultures. **(A)** Change in *Ifitm5* expression level. **(B)** Change in mineralization detected by alizarin red staining. **(C)** Change in the expression level of osteocalcin gene (*Bgla*p*2*). **(D)** Change in the expression of bone sialoprotein gene (*Ibs*p). n = 3. Data represent mean ± sd.

In osteoblasts isolated from mouse calvaria, 20 µg/ml FK506 considerably suppressed the expression of *Ifitm5* (Fig. 1A). Mineralization was also suppressed depending on the concentration of FK506 (Fig. 1B). In addition, the expression of mineralization markers osteocalcin (*Bglap2*) and bone sialoprotein (*Ibsp*) were also suppressed by 20 μg/ml FK506 (Fig. 1C,D). FK506 suppressed the expression of *Bglap2* and *Ibsp* as well as *Ifitm5*, however, it is unknown whether the expression of *Bglap2* and *Ibsp* is suppressed directly by FK506. Knockdown of *Ifim5* using *Ifitm5*-specific shRNA has been reported to suppress mineralization *in vitro*^2^. This suggests that the expression of the mineralization marker genes, *Bglap2* and *Ibsp*, may be dependent on the expression of *Ifitm5*. We propose that suppression of *Ifitm5* expression by FK506 causes suppression of mineralization, as in the case of *Ifitm5* knockdown, thereby resulting in a lower expression of *Bglap2* and *Ibsp*.

### Survival of mosaic mice depends on the mosaic ratio of the c.-14C > T mutation

In the in vitro experiment described above, FK506 suppressed the expression of *Ifitm5* and also suppressed mineralization. However, as bone formation in *Ifitm5*-deficient mice occurs almost normally^3,4^, expression of *Ifitm5* is dispensable for in vivo mineralization. In addition, it has been reported that the expression level of mineralization marker genes such as *Bglap* is increased in *Ifitm5*-deficient mice^4^. Therefore, if FK506 can suppress the expression of *Ifitm5* with the c.-14C > T mutation in vivo, it is may be assumed that a similar condition as *Ifitm5*-deficient mice is created, wherein normal bone formation occurs. Thus, heterozygous *Ifitm5* c.-14C > T mutant mice were created using Crispr/Cas9-based genome editing technology, and the effect of FK506 on the bone formation of mutant mice was investigated.

A mixture containing Cas9 protein, tracrRNA, crRNA, and ssODN was administered into fertilized eggs by electroporation. Of the mice born from these fertilized eggs, 63 survived (Table S1) and 11 exhibited a perinatal lethal phenotype (Table S2). Of the 63 mice that survived, 26 were male mice (Table S1).

For the selection of mosaic mice having a c.-14C > T mutation from the 26 male mice, DNA was extracted from the tail tip of these mice and subjected to genotyping. Of the 26 surviving male mice, 6 (F0-06, F0-15, F0-18, F0-31, F0-33, and F0-43) were mosaic mice with c.-14C > T mutation, and these 6 mice had a mosaic ratio of c.-14C > T mutation in the range of 25–50% (Table 1). Of the 6 mosaic mice, 4 mice (F0-06, F0-15, F0-18, and F0-33) had almost no mutation sequence other than c.-14C > T (Table 1). In contrast, mutation sequences with deletions of 9 and 22 bases (9D and 22D) in F0-31, and mutation sequences with insertion of 1 base (1I) in F0-43 were detected other than the c.-14C > T mutations (Table 1, Table S3).

**Table 1:** Percentage of each sequence detected in tail tip of 6 surviving mosaic mice.

	F0-06	F0-15	F0-18	F0-31	F0-33	F0-43
WT	69.12	61.45	70.23	2.51	48.06	48.32
c.-14C > T	27.95	35.32	26.97	25.05	49.08	38.94
9D	0	0	0	48.80	0	0
22D	0	0	0	22.33	0	0
1I	0	0	0	0	0	10.18

The sequences of WT, c.-14C > T, 9D, 22D, and 1I are shown in Table S3.

The 11 mice exhibiting a perinatal lethal phenotype were all mosaic mice with the c.-14C > T mutation (Table 2). The mosaic ratio of c.-14C > T mutation in these 11 mice was 48% or more, of which 4 mice (F0-D04, F0-D05, F0-D09, and F0-D10) had a mosaic ratio of 97%. In addition, a mutation sequence (c.-12G > A) other than c.-14C > T was found in F0-D07 (Table 2, Table S4). In these mosaic mice exhibiting a perinatal lethal phenotype, limb malformation, deformation of the ribs, and hypomineralized skull were observed (Fig. S1).

**Table 2:** Percentage of each sequence detected in the tail tip in 11 perinatal lethal mice.

	F0-D01	F0-D02	F0-D03	F0-D04	F0-D05	F0-D06	F0-D07	F0-D08	F0-D09	F0-D10	F0-D11
WT	6.03	49.10	32.66	0.19	0.16	48.48	18.44	48.37	0.16	0.16	48.36
c.-14C > T	89.10	48.26	64.03	97.21	97.18	48.89	62.66	49.06	97.37	97.20	48.58
c.-12G > A	0.01	0.06	0.03	0	0	0.04	16.17	0.03	0	0	0.06

The sequences of WT, c.-14C > T, and c.-12G > A are shown in Table S4.

The surviving mosaic mice were bred and the skeletons of 16-month-old mosaic mice F0-06 (mosaic ratio, 27.95%), F0-15 (mosaic ratio, 35.32%), F0-18 (mosaic ratio, 26.97%), and F0-31 (mosaic ratio, 25.05%) were observed. However, no remarkable skeletal abnormalities such as interosseous membrane ossification or hyperplastic callus formation that are characteristic features of OI type V were observed in these mosaic mice. (Fig. S2).

### Heterozygous c.-14C > T mutants exhibit a perinatal lethal phenotype

A study recently reported that heterozygous c.-14C > T mutant CD1 mice exhibited a perinatal lethal phenotype^24^. The surviving mosaic mouse, F0-33, had a 49.08% mosaic ratio of the c.-14C > T mutation (Table 1), and therefore the possibility of it being a heterozygote could not be excluded. Thus, the possibility of obtaining viable heterozygous c.-14C > T mutant mice by mating this mosaic mouse with WT mice was explored. Of the 40 mice born by mating F0-33 and WT mice, 30 mice (75%) survived, all of which were WT (Table S5). The other 10 mice (25%) exhibited a perinatal lethal phenotype and were all heterozygous mutants (Table S5). The percentage of heterozygous mutants born by mating F0-33 and WT was 25% which reflects the mosaic ratio of germ cells in F0-33. This suggests that F0-33 is not a heterozygous mutant.

Of the 34 mice born by mating a mosaic mouse, F0-43 with a mosaic ratio of 38.94% and WT mice, 16 mice (47.1%) survived and 18 mice (52.9%) exhibited a perinatal lethal phenotype (Table S5). In addition, of the 17 mice born by mating a mosaic mouse, F0-15 with a mosaic ratio of 35.32%, and WT mice, 14 mice (78.6%) survived and 18 mice (21.4%) exhibited a perinatal lethal phenotype (Table S5). It was also observed that of the mice born by mating the mosaic and WT mice, all the surviving mice were WT, and all those that exhibited the perinatal lethal phenotype were heterozygous mutants.

### Heterozygous c.-14C > T mutants have severe skeletal anomalies

**Figure 2:**
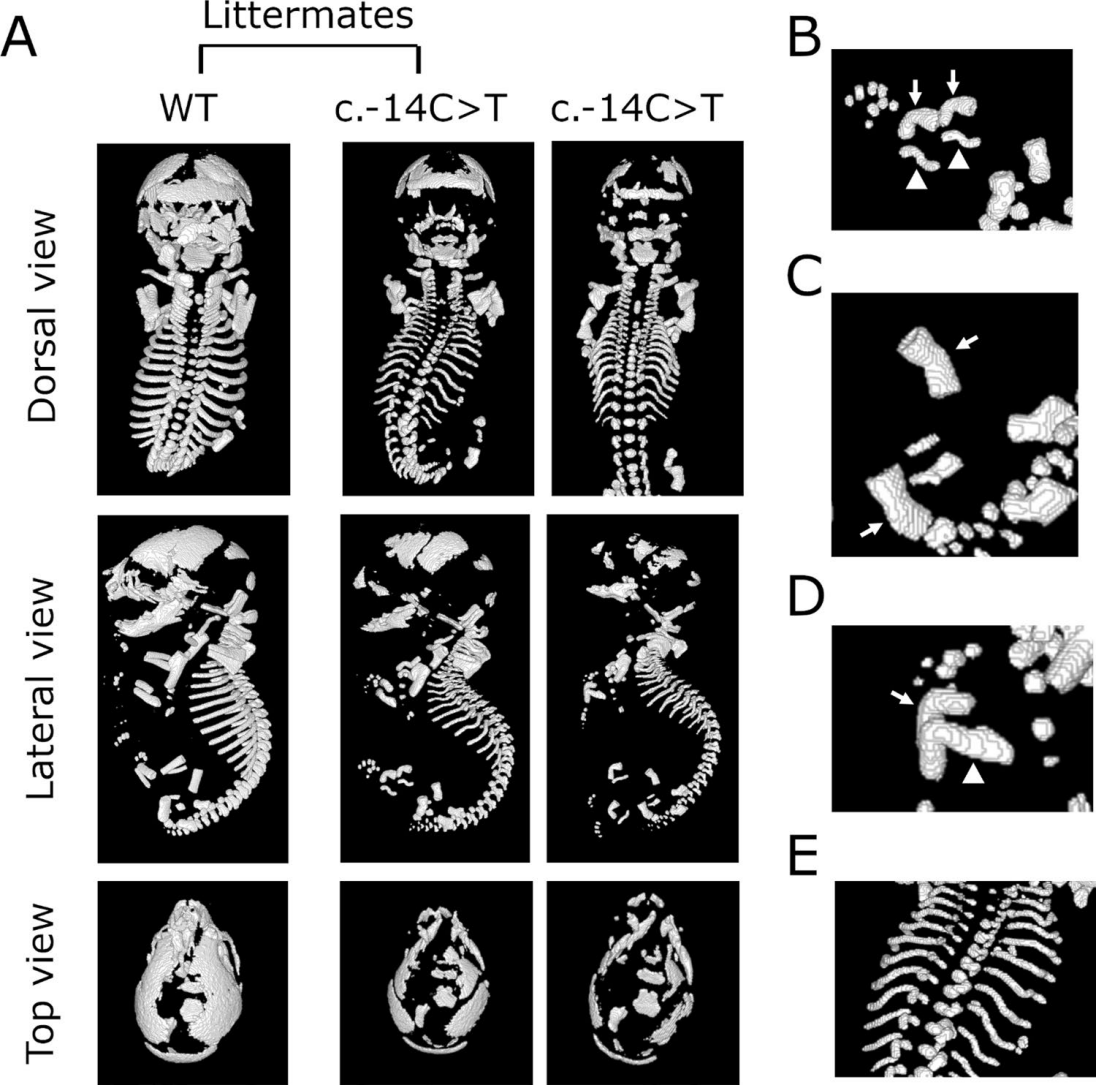
Representative micro-computed tomography (μCT) views of WT and heterozygous c.-14C > T mutant mice. **(A)** Dorsal (top), lateral (middle), and top (bottom) μCT views of neonates. WT in left panel and heterozygous mutant in middle panel were littermates. Neonate of heterozygous mutant shown in the right panel was born from a dam different from that of left and middle panels. **(B–E)** Anomalies of long bones and ribs of heterozygous mutant. Tibia (arrows) and fibula (arrowhead) **(B)**, femur (arrows) **(C**), radius (arrow) and ulna (arrowhead) **(D)**, and ribs **(E)** of heterozygous mutant.

In the heterozygous c.-14C > T mutants which exhibited a perinatal lethal phenotype, malformation of long bones and ribs and a hypomineralized skull were observed (Fig. 2A). In the hindlimbs of heterozygous mutants, the tibia was severely bent at the center, and the fibula was often bent twice. (Fig. 2B and Fig. S3). A gentle bending was also observed in the femur (Fig. 2C). In addition, the radius and ulna in the forelimbs were also severely bent in one place (Fig. 2D). Ribs of the heterozygous mutants were thin and wavy, and the rib cage tended to be smaller than that of WT (Fig. 2E, Fig. S4). The characteristics of these bone anomalies were the same as those observed in the mosaic mice exhibiting perinatal lethal phenotype with a mosaic rate of 48% or higher (Fig. S1).

The bone mineral content (BMC) of the neonates born by mating mosaic and WT mice was compared. The BMC in the whole skeleton of the heterozygous mutants was 42% of that of the WT mice (Table S6), whereas it was 37% in the ribs and thoracic vertebra and 38% in the skull (Table S6). These results indicate that skeletal mineralization in heterozygous mutants is markedly reduced.

### FK506 enhances mineralization in both heterozygous mutant and WT mice

The perinatal lethal phenotype exhibited by the heterozygous mutant mice prevented us from verifying the effect of FK506 after birth. Thus, to examine whether the administration of FK506 improves the skeletal anomalies of fetuses of the heterozygous mutants and allow these neonates to survive, FK506 was subcutaneously administered to WT pregnant dams that were mated with mosaic mice once a day from day 10.5 to day 18.5 of gestation.

**Figure 3:**
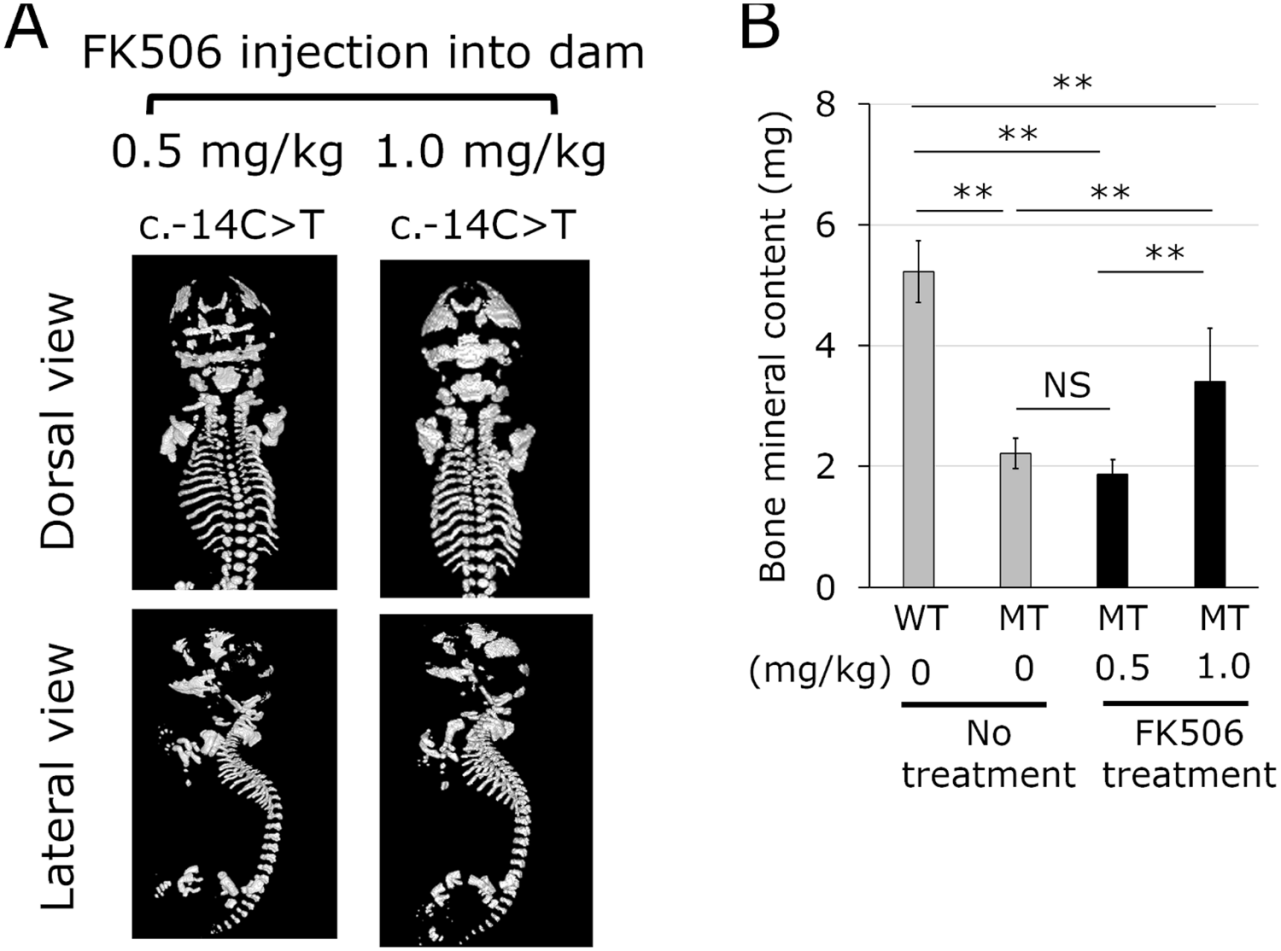
Representative micro-computed tomography (μCT) images and bone mineral content of neonates born from dams with FK506 treatment. A mosaic male mouse with the c.-14C > T mutation was mated with WT female mice. Pregnant dams underwent subcutaneous injection of FK506 with a dose of 0.5 or 1.0 mg/kg once per day from gestation day 10.5 to day 18.5. **(A)** μCT images of neonates of heterozygous c.-14C > T mutants treated with FK506 at doses of 0.5 and 1.0 mg/kg. **(B)** Bone mineral content of whole skeleton. WT, wild type; MT, heterozygous c.-14C > T mutant. Grey bar, bone mineral content in WT and MT mice without FK506 treatment. Black bar, bone mineral content in MT mice treated with FK506 at a dose of 0.5 and 1.0 mg/kg. Data represent mean ± sd. n = 6 in WT and MT mice without FK506 treatment. n = 5 and 10 in MT mice with FK506 treatment at a dose of 0.5 and 1.0 mg/kg, respectively. **, *p* < 0.01; NS, not significant.

In spite of administration of FK506 at a dose of 0.5 mg/kg to the pregnant dams, all neonates of the heterozygous mutants exhibited a perinatal lethal phenotype. These neonates had severe bone anomalies and showed no improvement in BMC in the whole skeleton (Fig. 3A,B). Next, the dose of FK506 was increased to 1 mg/kg. Even at this dose, all heterozygous mutant neonates exhibited a perinatal lethal phenotype and the abnormalities in the skeletal morphology did not improve (Fig. 3A). However, the whole skeletal BMC of mutant neonates treated with FK506 was 1.54 times higher than that of mutant neonates without FK506 treatment (Fig. 3B).

**Figure 4:**
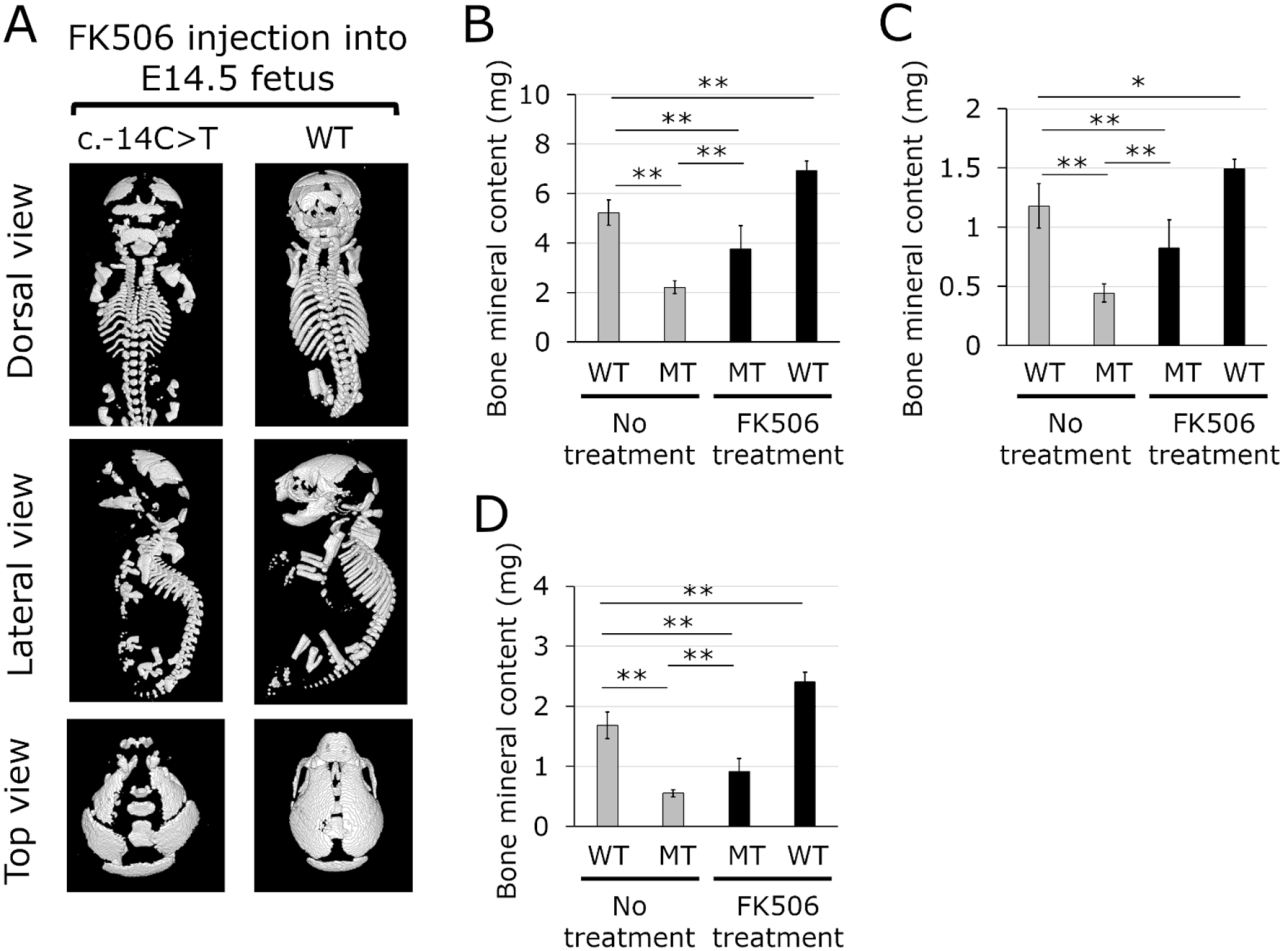
Representative micro-computed tomography (μCT) images and bone mineral content of neonates with FK506 treatment. A mosaic male mouse was mated with WT female mice. The E14.5 fetuses underwent transuterine intraperitoneal injection of FK506 at a dose of 1.0 mg/ml. **(A)** μCT images of neonates of a heterozygous c.-14C > T mutant and WT treated with FK506. **(B–D)** Bone mineral content. **(B)** Whole skeleton. **(C)** Ribs and thoracic vertebra. **(D)** skull. WT, wild type; MT, heterozygous c.-14C > T mutant. Grey bar, bone mineral content in WT and MT mice without FK506 treatment. Black bar, bone mineral content in MT and WT mice treated with FK506. Data represent mean ± sd. n = 6 in WT and MT mice without FK506 treatment. n = 6 and 4 in MT and WT treated with FK506, respectively. **, *p* < 0.01; *, *p* < 0.05; NS, not significant.

Although the mutant neonates showed an increase in BMC when the pregnant dams were administered with FK506 at a dose of 1.0 mg/kg, the abnormalities in skeletal morphology did not improve, implying that the c.-14C > T mutation containing *Ifitm5* continued to be expressed in the fetuses of the heterozygous mutants. Administration of FK506 directly into the peritoneal cavity of E14.5 fetuses through the uterus of WT pregnant dams mated with mosaic mice at an increased dose of 1 mg/kg increased the efficiency of FK506. However, even when FK506 was administered directly to the fetuses, all the neonates of the heterozygous mutants exhibited a perinatal lethal phenotype, and abnormalities of skeletal morphology did not be improve (Fig. 4A). Conversely, the whole skeletal BMC in the mutant neonates treated with FK506 was 1.69 times that of the untreated mutant neonates (Fig. 4B). The BMC, particularly in the ribs and thoracic vertebrae of the mutant neonates treated with FK506 increased 1.86 times that of the untreated mutant neonates (Fig. 4C, Fig. S5). The BMC in the skull of the mutant neonates treated with FK506 was also seen to be increased 1.66 times compared to that of untreated mutant neonates (Fig. 4D and Fig. S6).

Furthermore, an increase in BMC was also observed in WT neonates. The BMC in whole skeleton, ribs and thoracic vertebrae, and skull in WT neonates treated with FK506 was 1.32, 1.26, and 1.43 times that of untreated WT neonates, respectively (Fig. 4A–C, Fig. S5, S6). These results indicate that FK506 increases the BMC in both heterozygous mutants and WT mice.

The fact that direct administration of FK506 to the fetus failed to improve abnormalities of skeletal morphology suggests that FK506 did not suppress the expression of c.-14C > T mutation containing *Ifitm5.* This inference led us to assess the expression level of *Ifitm5* in the hind limb bones of the fetuses directly administered with FK506. Real-time quantitative polymerase chain reaction (qPCR) assay suggested that the expression of *Ifitm5* was not suppressed by FK506 administration (Fig. S7), although this result does not formally exclude the possibility of the suppression of mutant allele, because the qPCR assay did not discriminate between WT and mutant allele. Also, the expression levels of *Bglap2*, *Sp7*, and *Runx2* were not significantly altered (Fig. S7).

### Rapamycin reduces BMC in heterozygous mutants but not in WT mice

**Figure 5:**
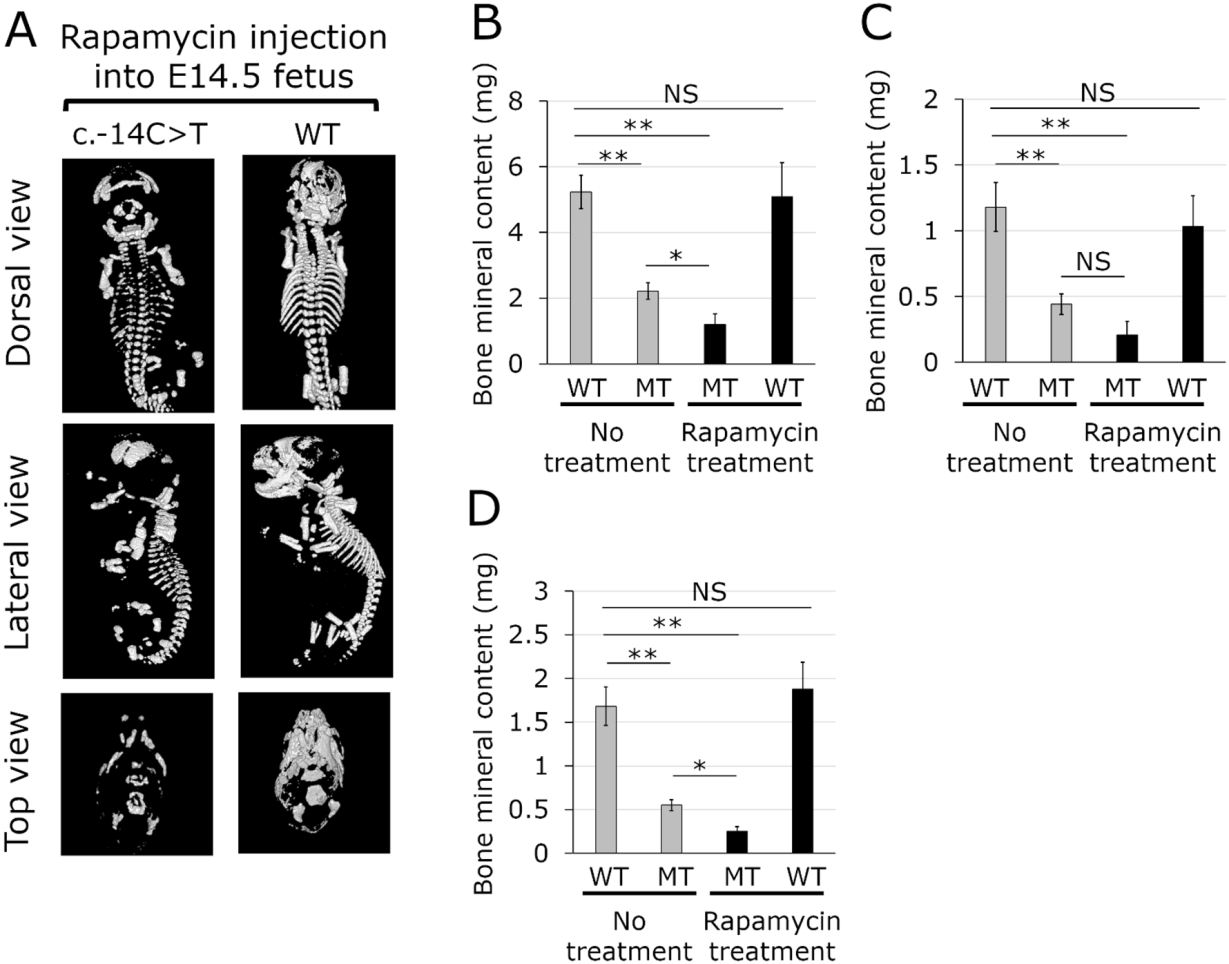
Representative micro-computed tomography (μCT) images and bone mineral content of neonates with rapamycin treatment. A mosaic male mouse was mated with WT female mice. The E14.5 fetuses underwent transuterine intraperitoneal injection of rapamycin at a dose of 1.0 mg/kg. **(A)** μCT images of neonates of a heterozygous c.-14C > T mutant and WT with rapamycin treatment. **(B–D)** Bone mineral content. **(B)** Whole skeleton. **(C)** Ribs and thoracic vertebra. **(D)** skull. WT, wild type; MT, heterozygous c.-14C > T mutant. Grey bar, bone mineral content in WT and MT mice without rapamycin treatment. Black bar, bone mineral content in MT and WT mice with rapamycin treatment. Data represent mean ± sd. n = 6 in WT and MT mice without rapamycin treatment. n = 7 in MT and WT with rapamycin treatment. **, *p* < 0.01; *, *p* < 0.05; *NS* not significant.

The effect of rapamycin, an immunosuppressant with a mode of action different from FK506, administered directly to the E14.5 fetuses was investigated to verify increased BMC in the neonates. Heterozygous neonates treated with rapamycin also exhibited a perinatal lethal phenotype and had severe skeletal anomalies (Fig. 5A, Fig. S8). This observation suggested that rapamycin does not suppress the expression of *Ifitm5* with the c.-14C > T mutation*.* The whole skeletal BMC in heterozygous neonates treated with rapamycin was reduced to 54% of that in untreated heterozygous neonates (Fig. 5B, Fig. S8). In contrast, the BMC of WT neonates treated with rapamycin did not differ from that of untreated WT neonates (Fig. 5B, Fig. S8). The BMC of ribs and thoracic vertebrae was reduced to 47% and that of the skull reduced to 46% in the mutant neonates treated with rapamycin as compared to the BMC in untreated mutant neonates; no significant difference was observed in the BMC of ribs and thoracic vertebrae between the rapamycin treated and untreated mutant neonates (Fig. 5C,D, Fig. S9, S10). The BMC of ribs, thoracic vertebrae, and skull in WT neonates treated with rapamycin was not significantly different from that of untreated WT neonates (Fig. 5C,D, Fig. S9, S10). These results suggested the possibility that the sensitivity to rapamycin in the heterozygous mutants is different from that in WT mice at least during embryogenesis.

## Discussion

Among the mosaic mice generated by Crispr/Cas9-based genome editing technology, mice with a mosaic ratio of 48% or more at the tail tip, with one exception (F0-33 with a mosaic ratio of 49.08%), exhibited a perinatal lethal phenotype, and severe bone abnormalities were observed. Mice with a mosaic ratio of 40% or less survived, and no skeletal abnormality was observed even after growth. These results suggest a relationship between mosaic rates and bone abnormalities. However, the relationship between the mosaic rate and bone abnormalities was not continuous, and it seemed that bone abnormalities appear above a certain threshold of mosaic rate. The mosaic rate was examined at the tail tip; although the mosaic rate of the tail tip does not reflect the mosaic rate of osteoblasts, it is proposed that abnormal bone formation is likely to occur in mosaic mice when the mosaic rate of osteoblasts exceeds a certain threshold.

Administration of FK506 suppressed the expression of *Ifitm5* in osteoblasts in vitro and at the same time suppressed mineralization. This result complied with the function of *Ifitm5* as a positive regulator of mineralization *in vitro*^2^ and also suggests the possibility of reduced mineralization in vivo. However, the absence of any significant abnormality in the skeleton of *Ifitm5*-deficient mice^3,4^ led to the possibility that mineralization would not be reduced if FK506 could completely suppress *Ifitm5* expression in vivo. In addition, it was considered that a complete suppression of both WT and mutated *Ifitm5* by FK506 may improve severe skeletal abnormalities in heterozygous c.-14C > T mutants. Based on this hypothesis, FK506 administration experiments were carried out. Neonates of heterozygous mutants obtained by direct administration of FK506 at a dose of 1 mg/kg to the fetuses showed improved BMC, but contrary to expectations, morphological bone abnormality did not improve suggesting that the expression of mutated *Ifitm5* was not suppressed. Assessment of *Ifitm5* expression in fetuses directly administered with FK506, revealed no suppression of *Ifitm5* expression. Therefore, the improvement in BMC seen after FK506 administration in heterozygous neonates is not due to suppression of *Ifitm5* expression. Although FK506 suppressed the expression of *Ifitm5 *in vitro, it did not suppress the expression of the gene in vivo. This indicates that the sensitivity of osteoblasts to FK506 is different in vitro and in vivo.

Direct administration of FK506 to the fetuses increased BMC not only in the heterozygous mutants but also in the WT mice, which indicates that FK506 has the same effect on heterozygous mutants and WT mice. In addition, an increase in BMC was observed in neonates of mutant mice even when FK506 was administered to the pregnant dams at a dose of 1.0 mg/kg. This result implies that FK506 administration to a pregnant dam also results in increased fetal BMC. FK506 forms a complex with the FK506-binding protein 12 (FKBP12)^25^, inhibiting the activation of the nuclear factor of activated T cells (NFAT), which is dependent on calcineurin^26^. NFAT functions as a co-factor for Sp1, Sp3, and Osterix, and activates these transcription factors^27,28^. Sp1, Sp3, and Osterix have been reported to be transcription factors that regulate the expression of *Ifitm5*^29^. The expression of *Ifitm5* in fetuses directly administered with FK506 suggests the activation of Sp1, Sp3, and Osterix in osteoblasts of fetuses with FK506 administration. The formation of a complex of FK506 with FKBP12 has been reported to dissociate FKBP12, an intracellular bone morphogenetic protein (BMP) repressor, from BMP receptor-1 (BMPR1), which activates BMP signaling^30^. BMP signaling activates Runt-related transcription factor 2 (RUNX2) which is an important transcription factor for differentiation of mesenchymal stem cells (MSCs) to immature osteoblasts^31^. Osteoclast-specific *Bmpr1* knockout mice suppress bone resorption, suggesting the involvement of BMP signaling in osteoclast activation^32^. Therefore, it can be concluded that activation of BMP signaling enhances both bone formation and bone resorption. In addition, phenotypes reported in osteoblast-specific or undifferentiated stem cell-specific *Bmpr1* conditional knockout mice^33–36^ suggest that the effect of BMP signaling on osteogenesis differs depending on the growth stage of mice and the differentiation stage of osteoblasts. The effect of the complex formation of FK506 with FKBP12 on osteogenesis is therefore considered to be complicated, and the effect cannot be easily estimated. To the best of our knowledge, there is no report suggesting that FK506 alone, without BMP or stem cells, enhances the mineralization of normal intact bones in mice. Although an increase in BMC was observed in neonates treated with FK506, continuous administration of FK506 subcutaneously to 4-week-old WT mice (C57BL/6 J) has been reported to inhibit bone formation^28^. It is unclear whether these contradictory results are due to differences in the growth stages of the mice or other causes. To improve bone mass and reduce fracture risk, bisphosphonates have been used as treatment for many types of OI, including type V^9^. The results of this study indicate that FK506 can be expected to have effects similar to bisphosphonates, at least during embryogenesis.

Direct administration of another immunosuppressant, rapamycin, to the fetuses did not affect the BMC of WT neonates but reduced the BMC of the heterozygous neonates. Similar to FK506, rapamycin forms a complex with FKBP12^37,38^, which inhibits the mammalian target of rapamycin (mTOR) signaling pathway^39,40^ unlike the FK506-FKBP12 complex which inhibits calcineurin. Rapamycin reduced the BMC of only the heterozygous neonates, suggesting that osteogenesis in the heterozygous mutants may be partially dependent on mTOR signaling. Recently, rapamycin has been identified as a candidate drug that suppresses ectopic ossification of progressive ossifying fibrodysplasia (FOP) ^41^, a condition in which activin-A binds to mutated ACVR1 (also known as ALK2), thereby activating BMP signaling^42,43^ and inducing ENPP2 production^41^. ENPP2 activates mTOR complex 1 signaling, resulting in aberrant chondrogenesis and subsequent induction of ectopic ossification^41^. The mechanisms of the interosseous membrane calcification and hyperplastic callus formation, which are characteristics of OI type V, are presently unknown. Although the initial phase of hyperplastic callus formation resembles an inflammatory reaction, the effectiveness of indomethacin, an anti-inflammatory agent, has not been observed in OI type V patients as reported in a previous study^44^. The result of this study indicating that the BMC of heterozygous mutants was reduced by rapamycin, which is involved in mTOR signaling may provide some clue to elucidate the mechanisms of interosseous membrane calcification and hyperplastic callus formation in OI type V.

In recent years, it has been reported that IFITM2 and IFITM3 that are potent inhibitors of virus-cell fusion and are degraded by rapamycin^45^. It has been found that the PPxY site (NEDD4 motif) is involved in this degradation, and IFITM1 without a PPxY site is not degraded by rapamycin^45^. It is expected that IFITM5 is not degraded by rapamycin, similar to because IFITM1, as IFITM5 does not have a PPxY site.

The perinatal lethal phenotype exhibited by the heterozygous c.-14C > T mutant mice led to an ambiguity as to whether the mutants exhibit characteristic symptoms of OI type V after birth. The skeletal phenotype of the heterozygous neonates, however, does not accurately reflect that of OI type V. This study has indicated that mineralization of heterozygous neonates may be partially dependent on mTOR signaling, and FK506 improves their BMC. As the skeletal phenotype caused by heterozygous mutation in IFITM5 differs between mouse and human, it is difficult to extrapolate the effects of FK506 or rapamycin on human OI type V. However, the findings obtained in this study are expected to enable elucidation of the mechanism by which MALEP-IFITM5 causes skeletal anomalies and provide essential information for development of therapeutic strategies.

## Materials and methods

### Osteoblast culture

Mouse skull-derived osteoblasts (# OBC11: Lot No. SBL C_OBM) were purchased from Cosmo Bio Co., Ltd. (Tokyo, Japan). The osteoblasts were dispersed in growth medium (OBCM, Cosmo Bio Co., Ltd.) at a density of 2.53 × 10^4^ cells/ml, and 500 μl of growth medium containing the dispersed cells was seeded in each well of a 24-well plate. After 5 days, the medium was changed to differentiation medium (OGCMO, Lot. SAI-DM-O, Cosmo Bio Co. Ltd) containing FK506 (FUJIFILM Wako Pure Chemical Corporation, Osaka, Japan) dissolved in dimethyl sulfoxide (DMSO) (FUJIFILM Wako Pure Chemical Corporation). The differentiation medium containing FK506 was replaced every 3 days. Detection of mineralized nodules was performed using Alizarin red S (FUJIFILM Wako Pure Chemical Corporation) after fixation of cultures by 70% ethanol.

### Generation of mosaic mice expressing *Ifitm5* containing c.-14C > T mutation

C57BL/6J mice were purchased from CREA Japan Inc. (Tokyo, Japan). Cas9 protein, crRNA-1, crRNA-2, tracrRNA, and single-stranded (ss) oligodeoxynucleotide (ODN) were purchased from Fasmac Inc. (Kanagawa, Japan). The nucleotide sequences of crRNA-1, crRNA-2, tracrRNA, and ssODN are listed in Table S7.

Superovulation was induced by intraperitoneal administration of CARD HyperOva (Kyudo Co., Ltd., Saga, Japan) to 4-week-old C57BL/6J female mouse, 48 h after which hCG (Asuka Pharmaceutical Co., Ltd., Tokyo, Japan) was intraperitoneally administered at 5 units/mouse. On completion of 15 h, an enlarged tube of the harvested fallopian tube was incised, and the ova were transferred to human tubal fluid (HTF) medium (ARK Resource Co., Ltd., Kumamoto, Japan). Spermatozoa were collected from the epididymal tail of a 10-week-old male mouse and incubated with in FERTIUP (mouse sperm pre-culture solution, Kyudo Co., Ltd.) at 37 °C for 1 h. In vitro fertilization was carried out by mixing the incubated spermatozoa with ovum in HTF medium. Five hours after in vitro fertilization, the mixture of Cas9 protein (final concentration of 100 ng/μl), tracrRNA (final concentration of 100 ng/μl), crRNA (final concentration of 200 ng /μl), and ssODN (final concentration of 200 ng/μl) was introduced into the embryos with male and female pronucleus by NEPA21 TypeII electroporator (voltage: 225 V, pulse width: 0.5 and 1.0 ms, pulse interval: 50 ms, number of times: 4 times; NEPAGENE Co., Ltd., Chiba, Japan). The electroporated embryos were transferred to KSOM medium (ARK Resources Co., Ltd.) and cultured for 16 h.

Transplantation of electroporated fertilized ova was done under anesthesia. Two-cell staged embryos after electroporation were transplanted into pseudo-pregnant mice on the day of confirmation of vaginal plugs. Birth of neonates was confirmed on the 19th day after embryo transplantation. DNA was recovered from the tail tip and the nucleotide sequence of the *Ifitm5* region was analyzed using the next-generation sequencer to investigate the mosaic ratio of the neonates. The mosaic mice described above were created at KAC Co., Ltd (Kyoto, Japan) with approval from the Animal Care and Use Committee of the Company.

### Generation of heterozygous *Ifitm5* c.-14C > T mutant mice

Male mosaic mice carrying the c.-14C > T mutation were mated with female wild-type (WT) C57BL/6J mice to obtain heterozygous mutants. Pregnant dams were bred by three methods until delivery. In the first method, pregnant dams were bred normally until delivery. In the second method, FK506 was administered subcutaneously to pregnant dams once a day from the gestation day 10.5 to day 18.5. In the third method, pregnant dams were anesthetized with isoflurane and surgically opened for direct administration of FK506 or rapamycin (LC Laboratories, MA, USA) from the uterus into the peritoneal cavity of E14.5 fetuses using a 33-gauge syringe. FK506 was administered to pregnant dams via subcutaneous injection (1.0 mg/ml in Soybean oil) at varying volumes depending on the concentration used [12.5 μl (0.5 mg/kg) or 25 μl (1.0 mg/kg)]. FK506 was administered to E14.5 fetuses [10 μl (1.0 mg/kg)] via intraperitoneal injection at a concentration of 0.03 mg/ml in phosphate buffered saline (PBS). Rapamycin, (0.5 mg/ml) was diluted in a solution containing 5% PEG400 and 5% Tween80 to a final concentration of 0.03 mg/ml with PBS and was administered 10 μl (1.0 mg/kg) to E14.5 fetuses via intraperitoneal injection.

Generation of heterozygous mutant mice described above were conducted at the Center for Promotion of Platform for Research on Biofunctional Molecules, Hokkaido University with approval from the Hokkaido University Animal Experiment Committee and were performed according to the guidelines for animal experimentation of the Hokkaido University.

### Determination of DNA sequence

The nucleotide sequence of the *Ifitm5* region in the mosaic mice was analyzed by a next generation sequencer. The tail tip of mice cut at 5 mm in length was placed in 300 μl of 25 mmol/l NaCl solution and incubated at 98 °C for 30 min to extract the DNA. The extracted DNA was purified with ethanol and then subjected to 1st PCR using ExTaq HS (Takara Bio Inc., Shiga, Japan). After purifying this PCR product with Agencourt AMPure XP (Beckman Coulter Inc., CA, USA), a second PCR was performed. The primer sequences used for the first and second PCR are given in Table S8. The obtained PCR product was purified with Agencourt AMPure XP and then the base sequence was analyzed with MiSeq (Illumina Inc., CA, USA) using MiSeq Reagent Kit v3 (Illumina Inc.).

The nucleotide sequence of the *Ifitm5* region in the heterozygous mice was analyzed by the Sanger sequencing method. The DNA extracted from the tail tip was amplified by PCR using forward (5′-CTGGTGGGTGGTCTCAAGCCACTGC-3′) and reverse (5′-AGGCAGCAACAGATTCAGGTACATCG-3′) primers. The base sequence of the amplified DNA was analyzed using Applied Biosystems 3730xl DNA analyzer (Thermo Fisher Scientific, MA, USA) with Applied Biosystems Big Dye Termination V3.1 (Thermo Fisher Scientific).

### X-ray micro-computed tomography (μCT) scanning and data analysis

An X-ray μCT apparatus (SMX-160CTS, Shimazu Corporation, Kyoto, Japan) was used for skeletal analysis. The mouse was wrapped in absorbent cotton, held vertically in posture, and set in the apparatus. The whole body of the mouse was scanned. It was confirmed that the CT value was not saturated in any of the samples. The X-ray μCT measurement of the standard substance (FANTOM No.06-U5D1mmH for bone mineral density, Ratoc System Engineering Co., Ltd., Tokyo Japan) was also performed under the same measurement conditions as the bone sample measurement. From the scanning results of the FANTOM, a regression line (R^2^ > 0.995) was obtained at 5 points for correspondence between bone mineral density (BMD; 100, 200, 300, 400, and 500 mg/cm^3^) and CT values, and used as a calibration curve. Extraction of bone area was performed by applying a 3D median filter with a radius of 2.0 pixels to remove minute noise after binarization with BMD 90 mg/cm^3^ as a threshold. Some bone areas were manually corrected as artifacts were observed. The bone areas of the skull, ribs and thoracic vertebra, tibia, and fibula (right hind limb) were manually created from the bone area of the whole skeleton. The bone volume was calculated from the number of pixels contained in the bone area and the pixel size. The total value of BMD was calculated from the total value of the corrected CT values of each pixel included in the bone area, and the bone mineral content (BMC) was determined by multiplying the volume per pixel.

### Gene expression analysis

In the extraction of nucleic acids from cultured osteoblasts, the cells were washed twice with PBS, then mixed with 800 μl of ISOGEN (Nippon Gene Co., Ltd., Tokyo, Japan), and were homogenized by pipetting. Nucleic acid extraction from the bones, the femur, tibia, and fibula was done using RNAlater (Thermo Fisher Scientific) after cutting the hindlimbs. These bones were placed in a disposable homogenizer (AS ONE, Osaka, Japan) containing ISOGEN and homogenized.

Chloroform was added to the homogenized cells and bones, followed by centrifugation at 12,000 × *g* for 15 min, and the supernatant was collected. An equal volume of 2-propanol was added to the supernatant, followed by centrifugation at 15,000 × *g* for 10 min to obtain nucleic acid pellet. The pellet was washed with 70% ethanol, dried, and dissolved in DEPC-treated water. DNA in DEPC-treated water was degraded with DNase I (Takara Bio Inc.), and then RNA was purified using Agencourt RNAClean XP (Beckman Coulter Inc., CA, USA) according to the manufacturer’s instruction. Purified RNA was reverse-transcribed with PrimeScript RT reagent Kit (Takara Bio Inc.) to obtain complementary DNA (cDNA). The light Cycler 480 SYBR Green I Master (Roche Life Science, IN, USA) was added to the synthesized cDNA, and the gene expression level was analyzed using qPCR (LightCycler 480, Roche Life Science). The primer sequences for qPCR of each gene are given in Table S9. The expression level of each gene was normalized with the expression level of *Gapdh*.

### Statistical analysis

All analyses were replicated with two to ten samples. Multiple comparisons were facilitated by assessment of significance using analysis of variance followed by Ryan’s method. A *p*-value < 0.05 was considered statistically significant.

## Supplementary Information

Supplementary Figures.

Supplementary Tables.
