# Supplementary material for: Effect of immunosuppressants on a mouse model of osteogenesis imperfecta type V harboring a heterozygous Ifitm5 c.-14C > T mutation
Source: Sci Rep. 2020 Dec 3;10:21197. doi: 10.1038/s41598-020-78403-1 (PMC7713238; doi:10.1038/s41598-020-78403-1)
Supplement: Supplementary file 1 — Supplementary Figures. [file 41598_2020_78403_MOESM1_ESM.pdf]

Supplementary Figures

Effect of immunosuppressants on a mouse model of osteogenesis imperfecta type V harboring a heterozygous *Ifitm5* c.-14C>T mutation

Nobutaka Hanagata[1,2*], Taro Takemura[1], Keiko Kamimura[3], Toshiaki Koda[3]

[1]Nanotechnology Innovation Station, National Institute for Materials Science, 1-2-1 Sengen, Tsukuba, Ibaraki 305-0047, Japan
[2]Graduate School of Life Science, Hokkaido University, N10 W8, Kitaku, Sapporo 060-0810, Japan
[3]Faculty of Advanced Life Science, Hokkaido University, N21 W11, Kitaku, Sapporo, 001-0021, Japan

*Corresponding author:
Nobutaka Hanagata
1-2-1 Sengen, Tsukuba, Ibaraki 305-0047, Japan.
Tel: +81-29-860-4774.
E-mail: HANAGATA.Nobutaka@nims.go.jp

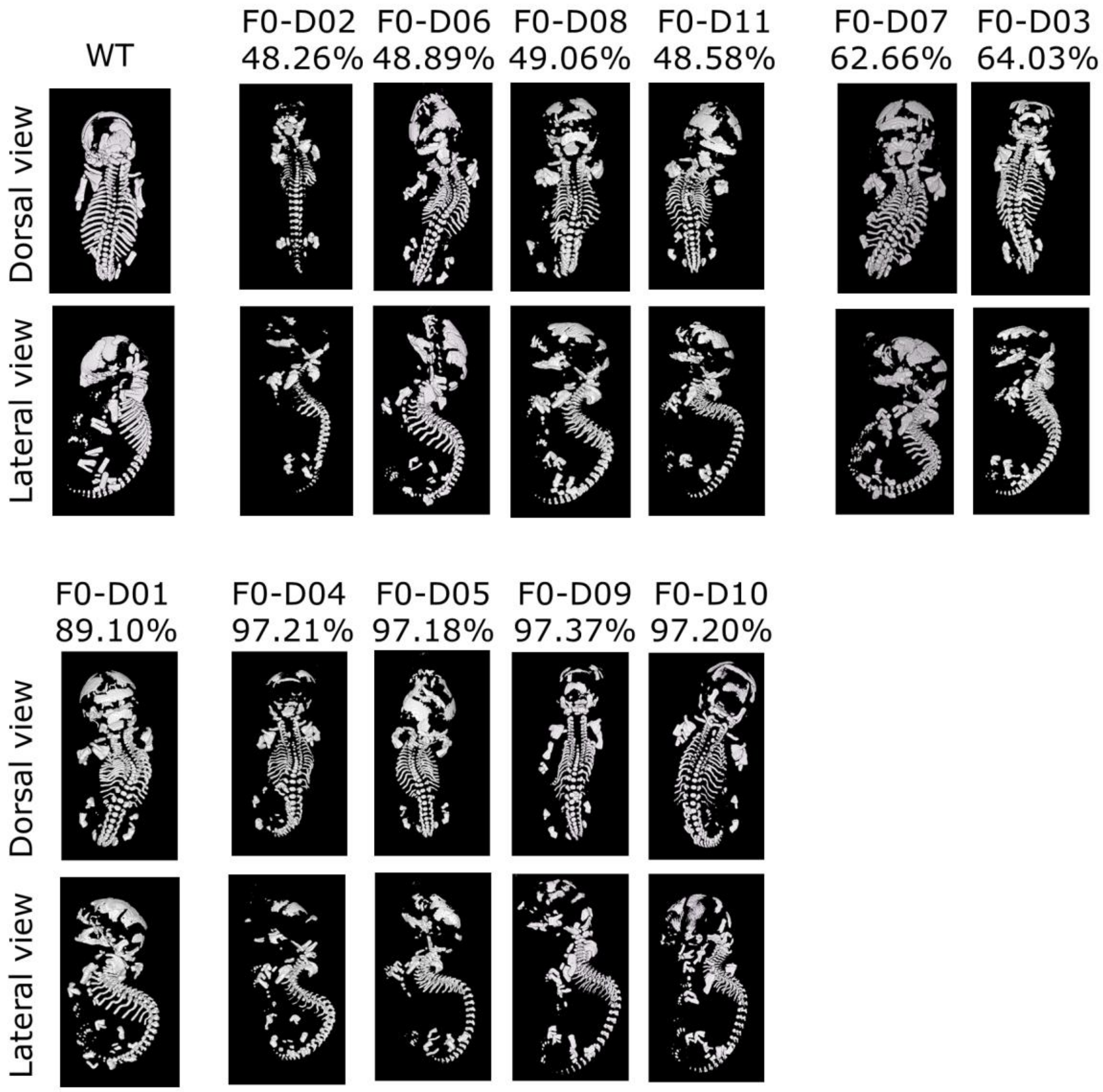

Fig. S1 Skeletal morphology of neonates of mosaic mice exhibiting lethal phenotype. WT, wild type. Description above image of dorsal view is individual identification number and mosaic ratio.

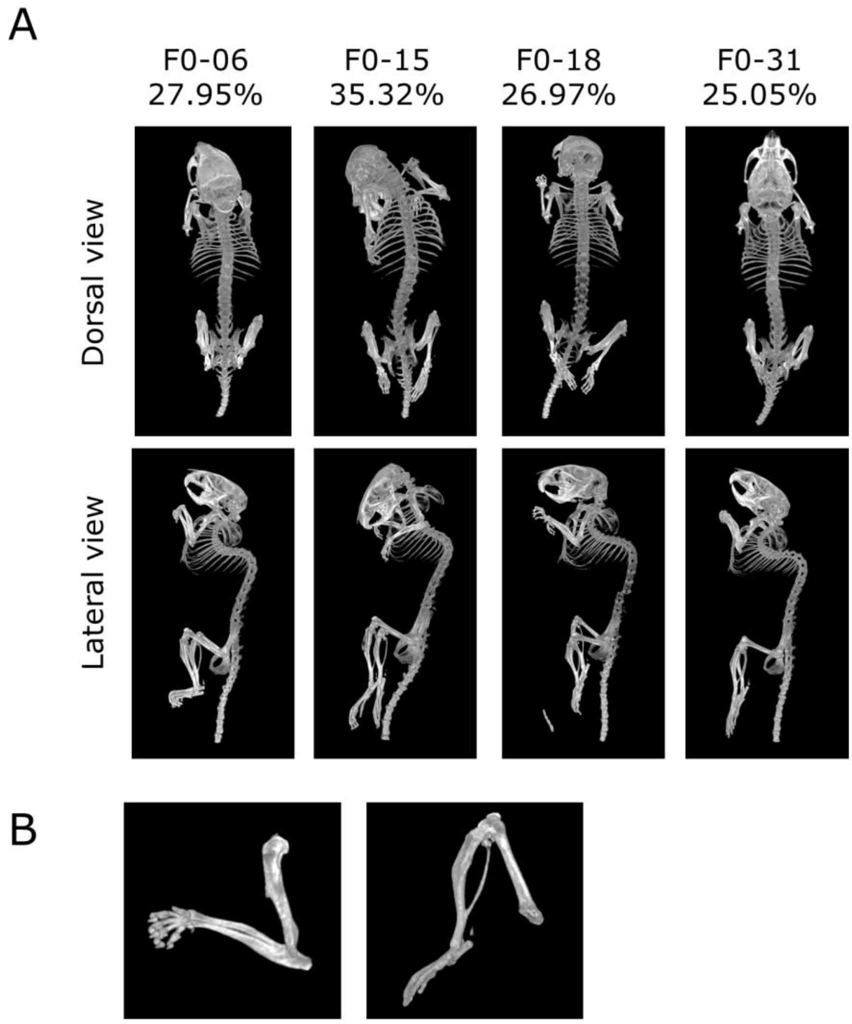

Fig. S2 μCT images of 16-month-old mosaic mice. (A) Whole skeleton. Description above image of dorsal view is individual identification number and mosaic ratio. (B) Forelimb (left panel) and hindlimb (right panel) of F0-18.

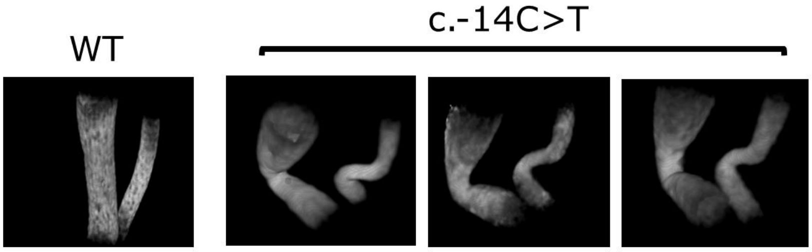

Fig. S3 μCT images of tibia and fibula in neonates of WT and c.-14C>T heterozygous mutants.

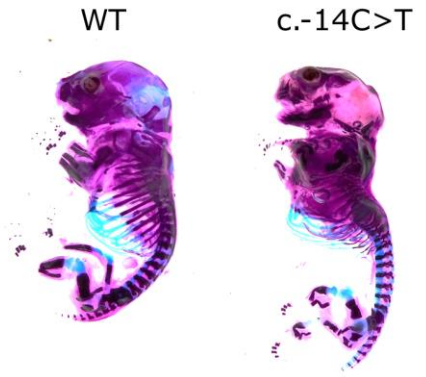

Fig. S4 Whole skeleton staining with alizarin red and alcian blue in neonates of WT and heterozygous mutant.

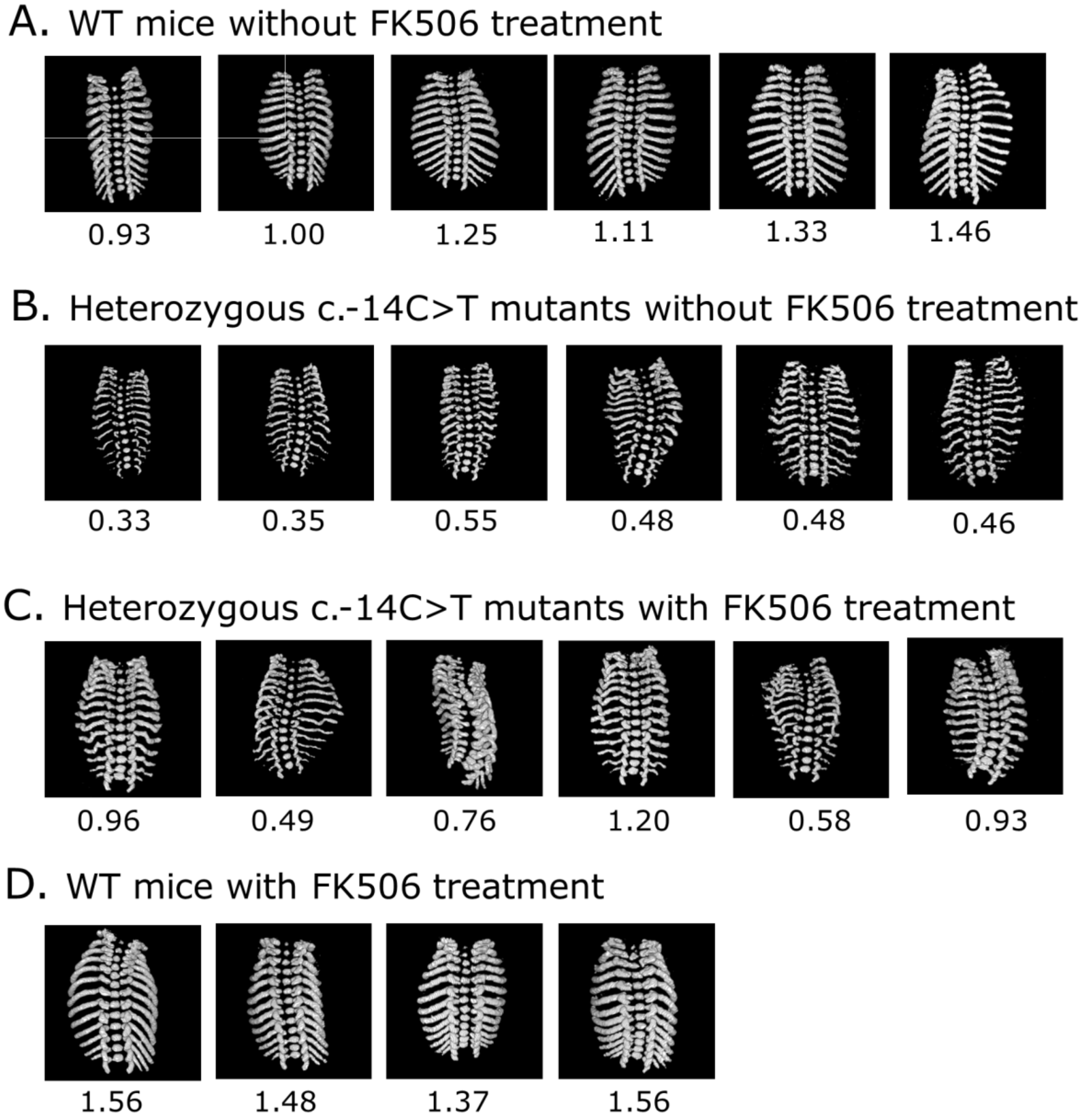

Fig. S5 Dorsal μCT views of ribs and thoracic vertebrae in neonates. The number described under the image is bone mineral content (mg).

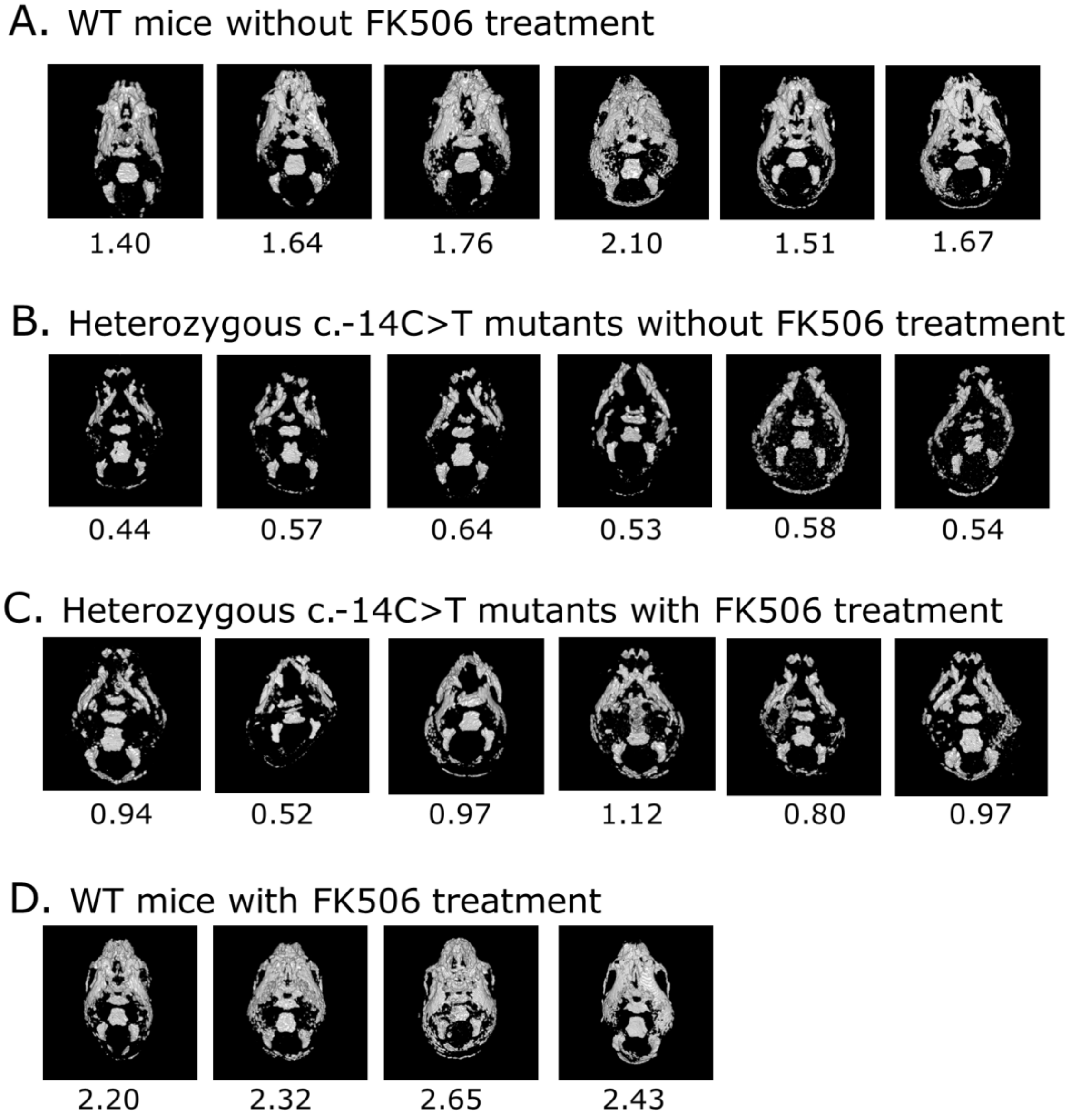

Fig. S6 Top μCT views of skulls in neonates. The number described under the image is bone mineral content (mg).

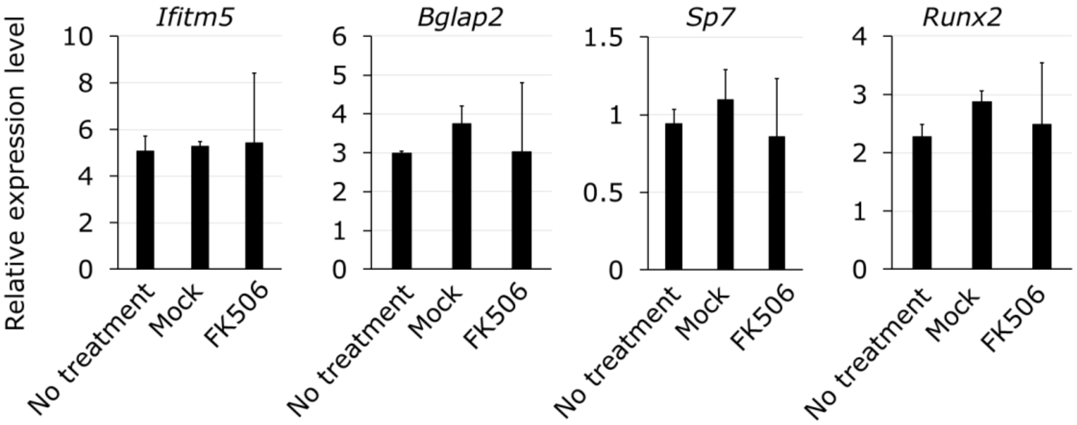

Fig. S7 Gene expression in E17.5 leg bones. In mock and FK506 treatment, the E15.5 fetuses underwent transuterine intraperitoneal injection of PBS (Mock) and FK506, respectively. n=2 in no treatment, n=3 in mock and FK506 treatment.

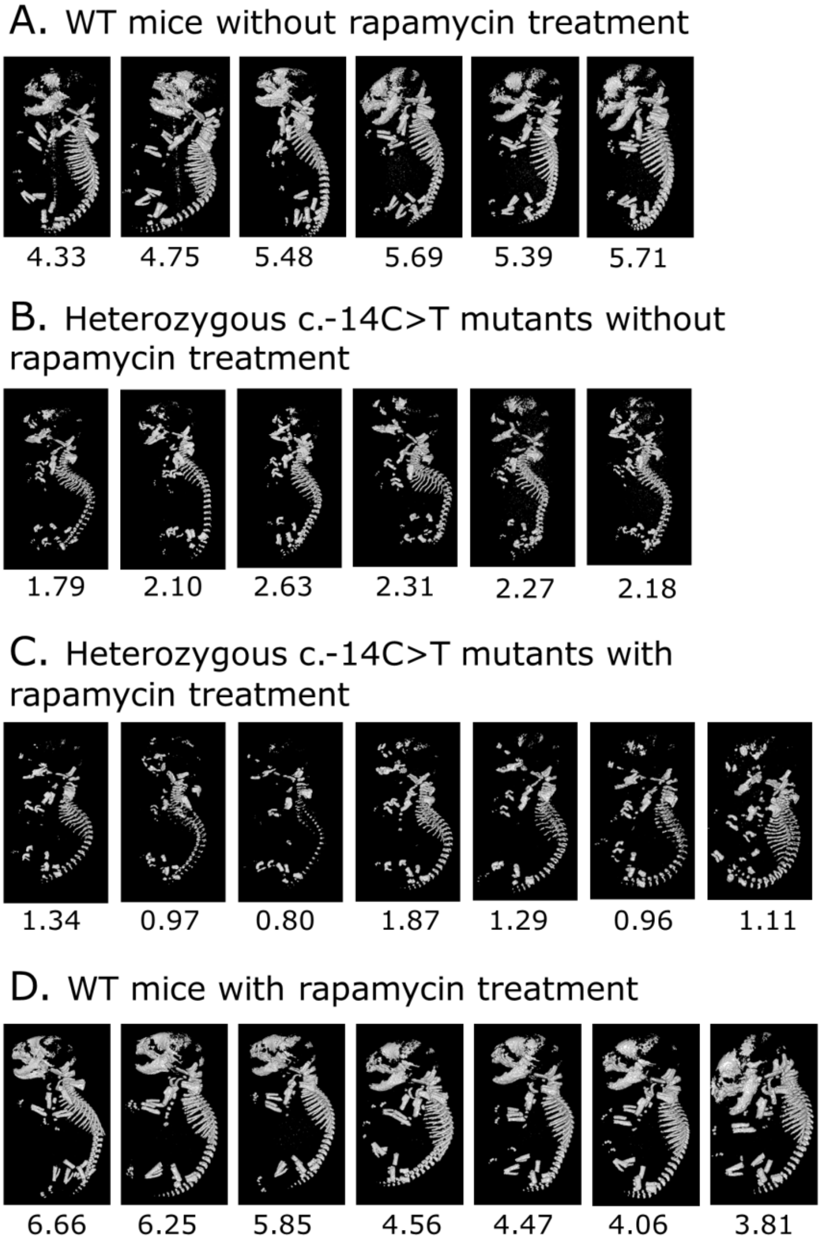

Fig. S8 Lateral μCT views of neonates without or with rapamycin treatment. The number described under the image is bone mineral content (mg).

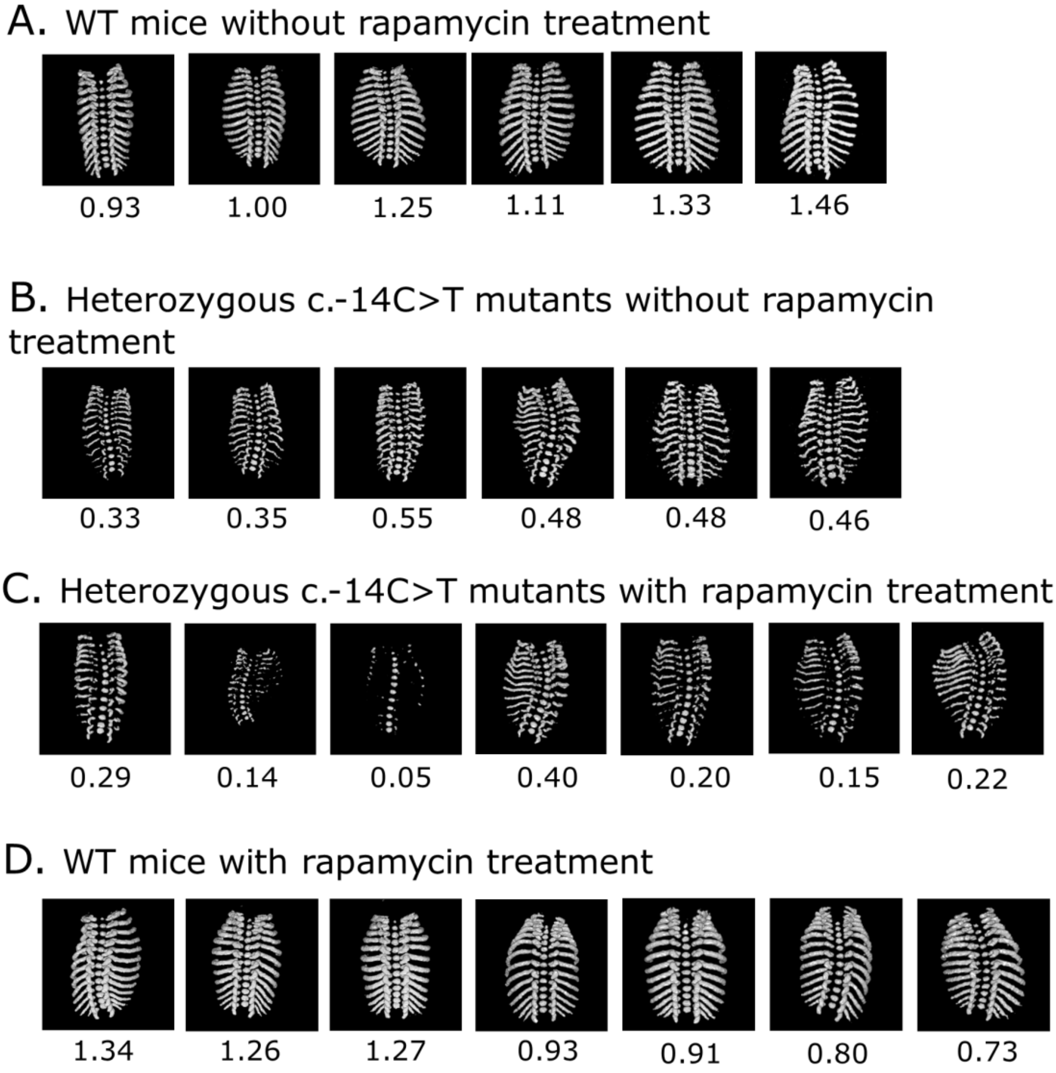

Fig. S9 Dorsal μCT views of ribs and thoracic vertebrae in neonates. The number described under the image is bone mineral content (mg).

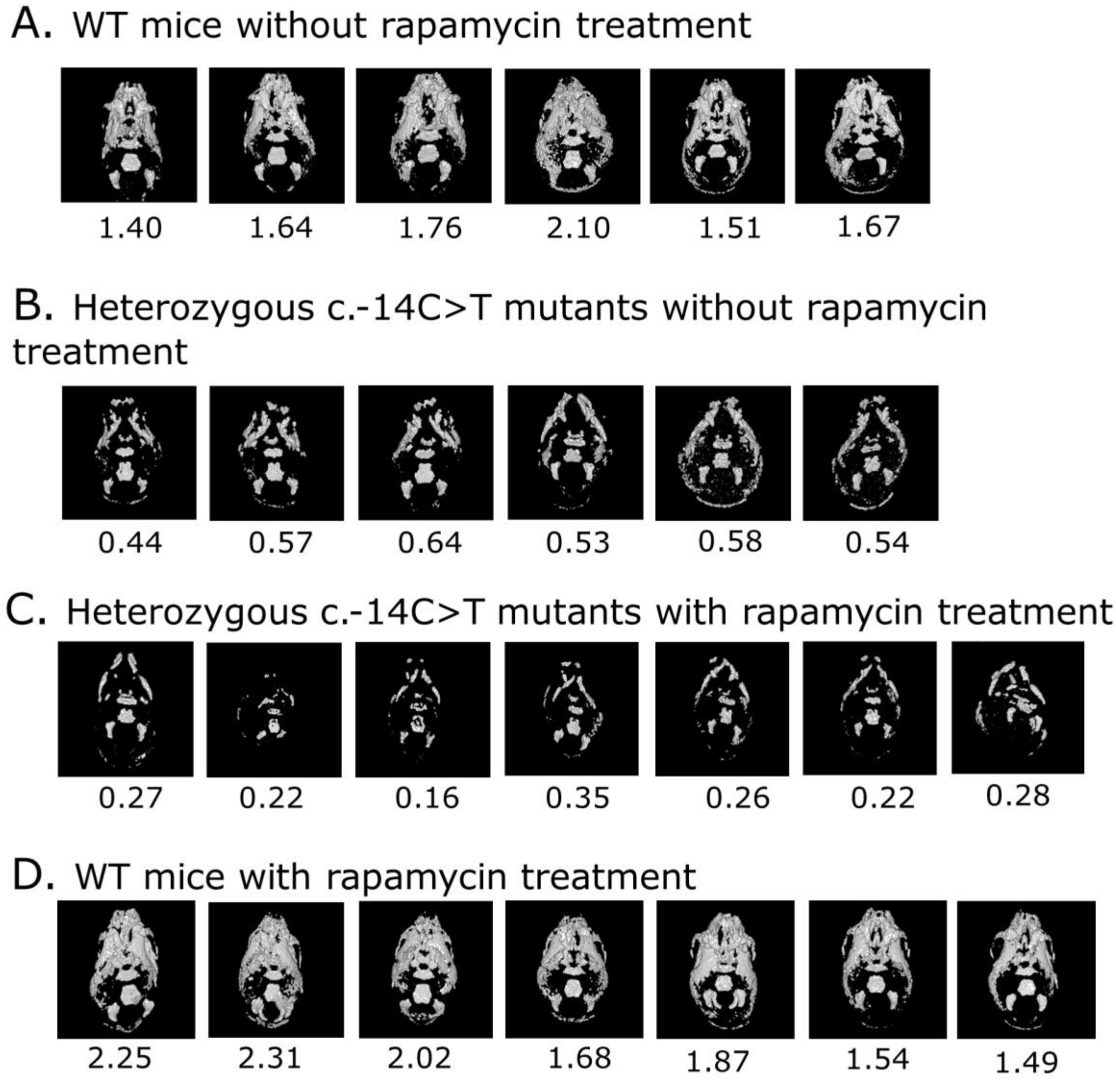

Fig. S10 Top μCT views of skulls in neonates. The number described under the image is bone mineral content (mg).
