# Supplementary material for: Effect of immunosuppressants on a mouse model of osteogenesis imperfecta type V harboring a heterozygous Ifitm5 c.-14C > T mutation
Source: Sci Rep. 2020 Dec 3;10:21197. doi: 10.1038/s41598-020-78403-1 (PMC7713238; doi:10.1038/s41598-020-78403-1)
Supplement: Supplementary file 2 — Supplementary Tables. [file 41598_2020_78403_MOESM2_ESM.pdf]

Supplementary Tables

Effect of immunosuppressants on a mouse model of osteogenesis imperfecta type V harboring a heterozygous *Ifitm5* c.-14C>T mutation

Nobutaka Hanagata[1,2*], Taro Takemura[1], Keiko Kamimura[3], Toshiaki Koda[3]

[1]Nanotechnology Innovation Station, National Institute for Materials Science, 1-2-1 Sengen, Tsukuba, Ibaraki 305-0047, Japan
[2]Graduate School of Life Science, Hokkaido University, N10 W8, Kitaku, Sapporo 060-0810, Japan
[3]Faculty of Advanced Life Science, Hokkaido University, N21 W11, Kitaku, Sapporo, 001-0021, Japan

*Corresponding author:
Nobutaka Hanagata
1-2-1 Sengen, Tsukuba, Ibaraki 305-0047, Japan.
Tel: +81-29-860-4774.
E-mail: HANAGATA.Nobutaka@nims.go.jp

Table S1. Number of surviving mice after birth

	Pulse width 0.5 msec [b)]	Pulse width 1.0 msec [b)]
crRNA-1[a)]	20[c)] (♂, 9; ♀, 11)	0
crRNA-2 [a)]	21[d)] (♂, 7; ♀, 14)	22[e)] (♂, 10; ♀, 12)

a) Type of crRNA used. The sequences of crRNA-1 and crRNA-2 are shown in Table S7.

b) Condition for electroporation

c) Born from 3 dams by embryo transfer

d) Born from 2 dams by embryo transfer

e) Born from 3 dams by embryo transfer

Table S2. Number of mice that died after birth

	Pulse width 0.5 msec [b)]	Pulse width 1.0 msec [b)]
crRNA-1 [a)]	0	11
crRNA-2 [a)]	0	0

a) Type of crRNA used. The sequences of crRNA-1 and crRNA-2 are shown in Table S7.

b) Conditions for electroporation

Table S3. Major nucleotide sequences identified from 6 surviving mosaic mice

**WT**	CTTGGTGCTCAGAGAGGACAAGTCTCAGCTAGGAAGACACGGCGCTGGAACCC-ATGGACACTTCATATCCCCGTGAGGACCCCCGGGCTCCATCATCCCGC
**c.-14C>T**	CTTGGTGCTCAGAGAGGACAAGTCTCAGCTAGGAAGACA**T**GGCGCTGGAACCC-ATGGACACTTCATATCCCCGTGAGGACCCCCGGGCTCCATCATCCCGC
**9D**	CTTGGTGCTCAGAGAGGACAAGTCTCAGCTAGGAAGACACGGCGCTGGA----------CACTTCATATCCCCGTGAGGACCCCCGGGCTCCATCATCCCGC
**22D**	CTTGGTGCTCAGAGAGGACAAGTCTCAGCTAGGAAGACATGGCGCTGGA-----------------------CCTGAGGACCCCCGGGCTCCATCATCCCGC
**1I**	CTTGGTGCTCAGAGAGGACAAGTCTCAGCTAGGAAGACACGGCGCTGGAACCC**C**ATGGACACTTCATATCCCCGTGAGGACCCCCGGGCTCCATCATCCCGC

Table S4. Major nucleotide sequences identified from mosaic mice that died after birth

**WT**	CTTGGTGCTCAGAGAGGACAAGTCTCAGCTAGGAAGACACGGCGCTGGAACCCATGGACACTTCATATCCCCGTGAGGACCCCCGGGCTCCATCATCCCGC
**c.-14C>T**	CTTGGTGCTCAGAGAGGACAAGTCTCAGCTAGGAAGACA**T**GGCGCTGGAACCCATGGACACTTCATATCCCCGTGAGGACCCCCGGGCTCCATCATCCCGC
**c.-12G>A**	CTTGGTGCTCAGAGAGGACAAGTCTCAGCTAGGAAGACACG**A**CGCTGGAACCCATGGACACTTCATATCCCCGTGAGGACCCCCGGGCTCCATCATCCCGC

Table S5. Genotype of mice generated from mating mosaic and WT mice

Mating	Offspring			
	Total number of birth	Survival or perinatal lethality	Ratio	Genotype
F0-33 x WT	40	Survival<br>Perinatal lethality	75%<br>25%	WT/WT<br>c.-14C>T/WT
F0-43 x WT	34	Survival<br>Perinatal lethality	47.1%<br>52.9%	WT/WT<br>c.-14C>T/WT
F0-15 x WT	17	Survival<br>Perinatal lethality	78.6%<br>21.4%	WT/WT<br>c.-14C>T/WT

Table S6. Bone mineral content in neonates of WT and heterozygous c.-14C>T mutant mice

	Whole skeleton (mg)	Ribs and thoracic vertebrae (mg)	Skull (mg)
WT mice[a)]	5.52±0.51	1.18±0.19	1.68±0.22
Heterozygous c.-14C>T mutants[b)]	2.21±0.25	0.44±0.08	0.55±0.06

a) n=6, mean±sd

b) n=6, mean±sd

Table S7. Sequences (5'→3') of tracrRNA, crRNA, and ssODN

tracrRNA	AAACAGCAUAGCAAGUUAAAAUAAGGCUAGUCCGUUAUCAACUUGAAAAAGUGGCACCGAGUCGGUGCU
crRNA-1<br>crRNA-2	CAGCUAGGAAGACACGGCGCGUUUUAGAGCUAUGCUGUUUUG<br>AGACACGGCGCUGGAACCCAGUUUUAGAGCUAUGCUGUUUUG
ssODN	CTATAAGCAGGCTTGGTGCTCAGAGAGGACAAGTCTCAGCTAGGAAGACA**T**GGCGCTGGAACCCATGGACACTTCATATCCCCGTGAGGACCCCCGGGCTC

Table S8 Primer sequences (5'→3') using PCR for the next-generation sequencing

	Forward	Reverse
1st PCR[a)]	acactctttccctacacgacgctcttccgatctCTGGTGGGTGGTCTACAGCCACTGC	tgactggagttcagacgtgtgctcttccgatctAGGCAGCACAGATTCAGGTACATCG
2nd PCR[b)]	AATGATACGGCGACCACCGAGATCTACACNNNNNNNNCACTCTTTCCCTACACGACGC	CAAGCAGAAGACGGCATACGAGATNNNNNNNNGTGACTGGAGTTCAGACGTGTG

a) Lower case letters for amplification with 2nd PCR

b) N is a tag sequence for identifying each sample

Table S9 Primer sequences (5'→3') for mouse gene analysis

Gene	Forward	Reverse
Gapdh	gtggacctcatggcctacat	gatggaaattgtgagggaga
Ifitm5	cagcgtcaacaccatcattc	cagaccagcagcactccata
Bglap2	atgaggaccctctctctgct	ccgtagatgcgtttgtaggc
Ibsp	tggaaggcattctttcttcaa	ggagacatttacttcctcctg
Sp7	tggagagggaaagggattct	gggctggggatcttagtgac
Runx2	cagaccagcagcactccata	cagcgtcaacaccatcattc
